# Study of Compaction Properties and Permeability Prediction of Multilayered Quadriaxial Non-Crimp Fabric in Liquid Composite Molding Process

**DOI:** 10.3390/polym12071525

**Published:** 2020-07-09

**Authors:** Yi Geng, Jinhua Jiang, Fangbing Lin, Huiqi Shao, Chenglong Zhang, Nanliang Chen

**Affiliations:** 1 Shanghai Collaborative Innovation Center for High Performance fiber Composites, College of Textiles, Donghua University, Shanghai 201620, China; gengyi@mail.dhu.edu.cn; 2 Engineering Research Center of Technical Textiles, Ministry of Education, Donghua University, Shanghai 201620, China; 2140024@mail.dhu.edu.cn (F.L.); hqshao@dhu.edu.cn (H.S.); zhangcl1995she@163.com (C.Z.)

**Keywords:** enhanced permeability, preform compaction, unit cell, numerical simulation, vacuum-assisted resin transfer molding

## Abstract

A systematic experimental study was performed to detect the compaction and permeability properties of multilayered biaxial and quadriaxial preforms under vacuum pressure. Compression response on ply level showed that the degree of nesting between quadriaxial NCF was more pronounced and the nesting deformation mechanism was affected by the interaction with stitch yarns. Owing to the meso-channels in the fibrous structure and the nesting between layers, the in-plane permeability of quadriaxial NCF did not follow an inverse proportion relationship with the fiber volume fraction. To predict the in-plane permeability of multilayered quadriaxial NCFs, unit cell models at a high level of geometrical details were built, including local variations in yarn cross-sections and the nesting deformation between layers. Numerical methods were implemented, and the prediction results were in very good agreement with the experimental data. Besides, the major contributing parameters to the enhancement of the in-plane permeabilities were identified by investigating the correlation between permeability and structural parameters of quadriaxial NCF. The modeling methodology and the principles established can be applied to the design of the quadriaxial NCF fabrics, where the permeability enhancement was evidenced.

## 1. Introduction

Non-crimp fabrics (NCFs) are widely used as textile reinforcement for composite materials. NCFs are built up by layers of fiber bundles that are stitched together by warp-knitting loops in different directions through thickness. Since NCFs show better axial mechanical properties and have much higher production efficiency than other crimped textile reinforcements, NCFs are especially applied in liquid composite molding (LCM) processes as reinforcements for large and thick composite parts. LCM is a widely used technique for the manufacturing of composite parts with large sizes, complex shapes, short cycle time and low cost. It includes subclasses like resin transfer molding (RTM) and vacuum-assisted RTM (VARTM). LCM techniques have in common that a stack of fabric layers is placed into a mold and a liquid resin is subsequently driven through the textile reinforcement. The resin impregnation process is driven by an injection pressure and the solid composites formed after the resin cures. The process of the liquid resin flowing through fibrous reinforcements is described by Darcy’s law (Equation (1)), which states a dependence of the flow velocity on the permeability of the medium, viscosity of the liquid and the applied pressure gradient [1,2,3,4].

(1) $$v=-\frac{K}{\mu }\cdot \nabla P$$

where *v*, *µ*, ∇*P* and *K* are the volume averaged velocity, the dynamic fluid viscosity, the pressure gradient across the porous medium and the permeability, respectively. The permeability of fibrous preform is a measure of the resistance that a porous medium opposes to fluid flow. Knowledge of the permeability allows the estimation of the processing time and evaluates the robustness of the process [5,6,7]. Thus, an accurate value for the permeability of the reinforcement is highly required in the molding processes of composite materials.

Permeability can be determined by experiment measurements or modeling approaches. Measuring permeability is time and resource-consuming and the values obtained with different experiments or set-ups suffer from large scatter [8,9,10,11], while permeability prediction has the potential to help with these issues [12]. The predictive permeability modeling approaches depend on the geometric characteristics of a given porous medium. The fiber orientations, bundle shapes and dimensions, even the stitch yarn of the preform could lead to significant permeability variations [8,13,14,15,16,17]. Hence to compute the permeability of a NCF, a detailed geometrical model of the textile is required. For instance, Endruweit et al. analyzed axial duct flow through gaps between adjacent yarns. It is illustrated that at a certain fiber volume fraction and yarn spacing, the maximum and minimum values for the equivalent permeability of inter-yarn gaps differ by factors of up to three [13]. Besides, Martin et al. found that in quasi-unidirectional non-crimp fabrics, a reduction of the stitch length improved the global permeability and a loose stitch increased the in-plane transverse permeability [14]. Loendersloot et al. proposed that the effective permeability of biaxial NCFs depends on the wedge-shaped channels induced by stitch yarns penetrating the fabric [16]. And, Syerko et al. studied the link between the permeability and the geometrical parameters of unidirectional NCF fabric with a weft backing layer, identifying the major contributing parameters to the enhancement of the in-plane and through-thickness permeability [17]. Specifically, NCF reinforcements are deformed when they are compacted into the mold during the preforming process. For multilayered preforms, the interaction between adjacent fabric layers intensifies and the tows in adjacent fabric layers push each other, which directly affects the global permeability [12,18,19,20,21,22,23,24,25,26,27]. Therefore, permeability computation methods have related the geometry models to the geometrical perturbations due to deformation. For instance, Haanappel et al. developed a network flow model for ±45° biaxial NCF, considering the external channels due to the push of the stitch yarns into fibers at the compression [27]. Zeng et al. provided unit cell models for 3D-orthogonal weave reinforcement, including yarn cross-section variations and variability in yarn paths under realistic manufacturing conditions [20]. With inclusion of local variations in geometrical modeling, the predictions of fabric permeability significantly improved compared with the experimental data.

However, most investigations have been limited to biaxial NCF, which is made up from unidirectional tows orientated from 0°/90° or +45°/−45°, neglecting other NCFs that with different fiber orientations. For example, quadriaxial NCF, analysts usually regarded the quadriaxial NCF as four layers of unidirectional tows stacks together with no further detailed description [21], which results in inaccurate approximations of the permeability. Actually, quadriaxial NCF, which makes up from not only unidirectional tows orientated from 0°/90° but also weft backing fiber piles orientated from +45°/−45°, It has been revealed that the compaction property, nesting behavior between layers as well as the permeability properties are quite different from the biaxial NCFs [28,29,30,31,32,33,34,35]. As the quadriaxial NCF is also a widely used reinforcement in LCM, it is of great significance to characterize the compaction and nesting behavior of the textile in the molding process and proposes an accurate permeability prediction model for the preforms.

In this paper, the compression and permeability properties were studied for both biaxial and quadriaxial preforms in VARTM to investigate the relationship between the compaction deformation, nesting behavior and permeability for the two NCFs. Based on the experiment results, a modeling approach based on geometrical variations of multi-orientated tows and self-imposed kinematic constraints of stitch yarns was developed for multilayered quadriaxial preforms. According to the geometry of the fibrous structure, the permeability prediction model for the multilayered quadriaxial NCF was proposed to help with the design of composite reinforcements. The correlation between the permeability and the structural parameters of the quadriaxial NCFs was investigated numerically and the major contributing parameters to the enhancement of the in-plane permeability were identified. The principles established in this study were applied to the design of quadriaxial preform in VARTM, where the permeability enhancement was evidenced.

## 2. Experiments

### 2.1. Material

**Figure 1:**
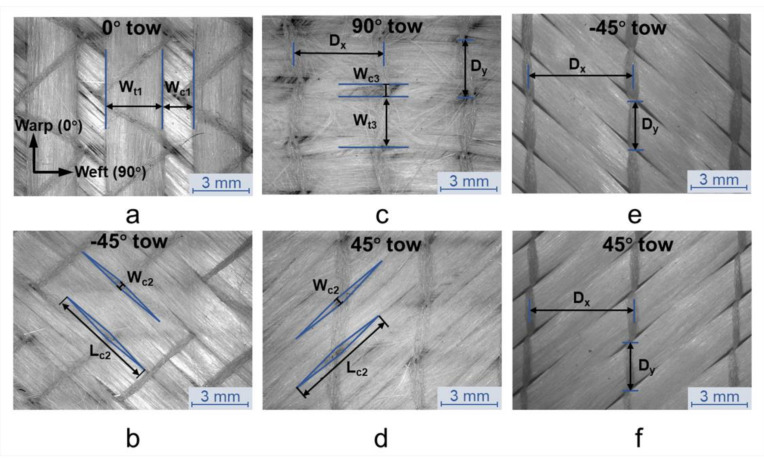
(**a**) Structure of fiber tows orientated at 0° in quadriaxial NCF, E-DBLT800-6^TM^; (**b**) structure of fiber piles orientated at −45° in quadriaxial NCF, E-DBLT800-6^TM^; (**c**) structure of fiber tows orientated at 90° in quadriaxial NCF, E-DBLT800-6^TM^; (**d**) structure of fiber piles orientated at 45° in quadriaxial NCF, E-DBLT800-6^TM^; (**e**) structure of fiber tows orientated at −45° in biaxial NCF, E-DB800^TM^; (**f**) structure of fiber tows orientated at 45° in biaxial NCF, E-DB800^TM^.

To investigate the compression and permeability properties of quadriaxial NCF, compression experiments and permeability tests are carried on two types of glass fabric supplied by PGTEX China Co., Ltd.: a quadriaxial NCF, E-DBLT800-6^TM^ with areal weight 821 g/m^2^ and a biaxial NCF, E-DB800^TM^ with areal weight 813 g/m^2^ for comparison. E-DBLT800-6^TM^ is knitted basing on the two piles of unidirectional tows (0° and 90°) and stabilized by two weft backing layers orientated at ±45°, while E-DB800^TM^ consists of two plies of fiber tows orientated at ±45°. The geometry of the two NCFs is shown in Figure 1. And the stitch distance (*D_x_*) and the stitch length (*D_y_*) of the stitch pattern for the two fabrics are marked in Figure 1c,e. Properties of the two NCFs are reported in Table 1, Table 2 and Table 3. It is noted that the two NCFs have the similar areal weight and the stitch distance, implying that the only difference between the two NCFs is the fibrous structure.

### 2.2. Compaction Testing Setups

Fabric compaction tests were performed on a single layer, as well as on stacks of two, four and six layers with the same orientation (0°) for biaxial and quadriaxial NCFs (Table 4). Preforms of the same orientation and layups were sealed and vacuumed in the VARTM setting (one side is vacuum bag and another side is metal tool). Then the VARTM setting was placed in room temperature for more than 12 h before the total thickness of the compacted preforms was measured. Ten measurements were made at each test and the total thickness was taken as the compaction thickness of the preform under vacuum (*H_V_*). Then resin was infused with vacuum pressure using the VARTM technique and after the resin cured, the thickness of laminates (*H_L_*) was recorded as the thickness when resin impregnated the dry preform. The *H_L_* value of each preform was used to estimate the fiber volume fraction for the permeability calculation. Also, the average thickness per layer (*H_VP_* and *H_LP_*) was calculated to characterize the compaction mechanism and nesting behavior in different compaction states.

### 2.3. Permeability Testing Setups

**Figure 2:**
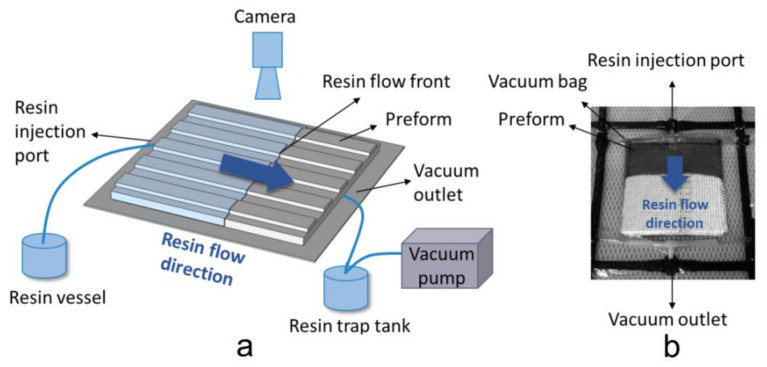
(**a**) Scheme of the unidirectional in-plane permeability test setup; (**b**) rein flow pattern during the in-plane permeability test.

Permeability measurements were conducted with a unidirectional permeability test setup using the VARTM technique (Figure 2). In the experiments, the same preform styles were used in each experiment (Table 4). Before the resin infusion, the preform was sealed and vacuumed in the VARTM setting and placed in room temperature for more than 12 h. A type of unsaturated polyester (HS-2104-G40, 320 cp, Changzhou Huake Polymers Co. Ltd., Changzhou, China) impregnated the fabric layers from one side to the other side, keeping a one-dimensional flow with very small race-tracking effect around the edges. The resin impregnation process was driven by a vacuum pump and a digital video camera was employed to monitor the flow front position vs. time in each experiment. Three measurements were made at each test and the in-plane unidirectional permeability was calculated from experimental data according to the squared flow front (SFF) method [9].

## 3. Experiment Results and Discussion

### 3.1. Compaction Response of Single and Multilayered Preforms

**Figure 3:**
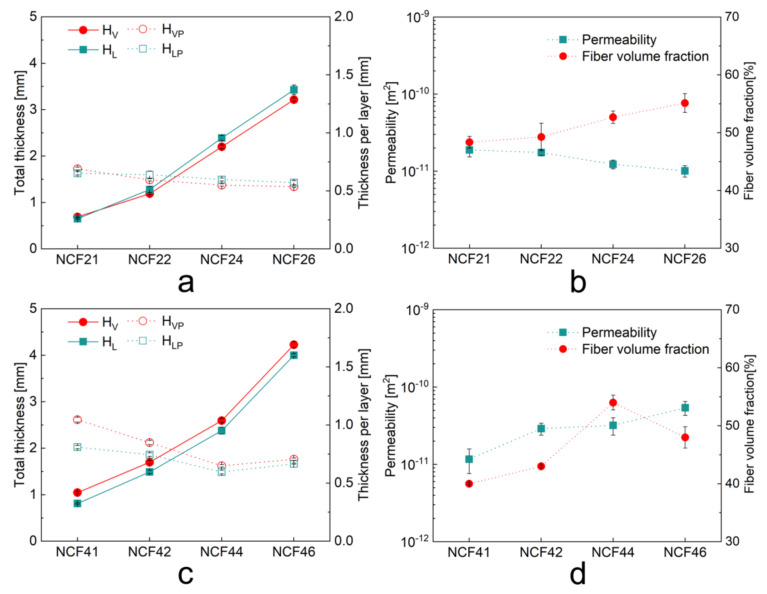
(**a**) Total thicknesses and average layer thicknesses of the biaxial preforms at varied compaction states; (**b**) unidirectional permeability and fiber volume fraction of biaxial preforms with different numbers of layers; (**c**) total thicknesses and average layer thicknesses of the quadriaxial preforms at varied compaction states; (**d**) unidirectional permeability and fiber volume fraction of quadriaxial preforms with different numbers of layers.

The total thickness and the average layer thickness of the biaxial and quadriaxial NCFs based preforms under vacuum pressure is plotted in Figure 3a,c. In Figure 3a, an increase in the thickness of multilayered biaxial preforms appears from dry to wet state (from *H_V_* to *H_L_*), resulting in a decrease in the fiber volume fraction after the resin impregnation. While in Figure 3c, a decrease in the thickness of all the quadriaxial preforms is noticed from dry to wet state (from *H_V_* to *H_L_*). This phenomenon demonstrates that during the post-filling period, as the resin pressure inside the laminate decreases and equilibrates, the compaction mechanism of biaxial and quadriaxial NCFs is varied. For biaxial preforms, the thickness per layer slightly decreases first and remains constant as the layer number increases, which shows very rare nesting deformation between layers. That is because the stitching yarn goes across the tows on both surfaces of the biaxial NCF (Figure 1a,b) and it works as a web to prevent the nesting altogether. While for quadriaxial based preforms, the thickness per layer decreases obviously before the layup reaches four. After that, a slight increase is noticed from a four-layer stack to a six-layer stack. Apparently, the nesting is significant since the quadriaxial NCF has wider channels between tows, leaving enough space for the tows on the backside of the adjacent layer to nest inside the channel (Figure 1c–f). Moreover, the tricot stitch on the surface of quadriaxial NCF goes across the 0° tows and the remaining part of the stitch penetrates other fiber piles. As a result, when stacked in a multilayered preform, the remaining part of the stitch could be stretched by other fiber piles with significant nesting deformation while the stitch on the surface is forced to shrink along the weft direction. Since the fibers inside tows have already been compactly packed from a four-layer stack to a six-layer stack, it is speculated that the above reason causes the increase of layer thickness, which will be verified further by the analysis of internal geometries of laminates.

### 3.2. Permeability of Single and MultiLayered Preforms

In Figure 3b, the permeability data and fiber volume fraction of biaxial based preforms are presented. For biaxial preforms, the fiber volume fraction increases and the permeability decreases as the increases of layers. That is because the cross-section of meso-channel between tows and the space between fibers inside the tows is reduced as the layer increases. In contrast, for quadriaxial preforms (Figure 3d), both the permeability and the fiber volume fraction increase before four layers. Afterwards, the permeability keeps increasing while the fiber volume fraction decreases from a four-layer stack to a six-layer stack. Besides, it is noted that the permeability rises at a decreasing rate before four layers while the permeability rapidly increases from a four-layer stack to a six-layer stack. It implies that the permeability of quadriaxial preforms is mainly affected by the meso channels between tows instead of the fiber volume fraction. And, a rapid augment of flow channels along the flow direction from a four-layer stack to a six-layer stack is expected, which will be further verified by the analysis of internal geometries of laminates.

### 3.3. Internal Geometry of Single and Multilayered Preforms

**Figure 4:**
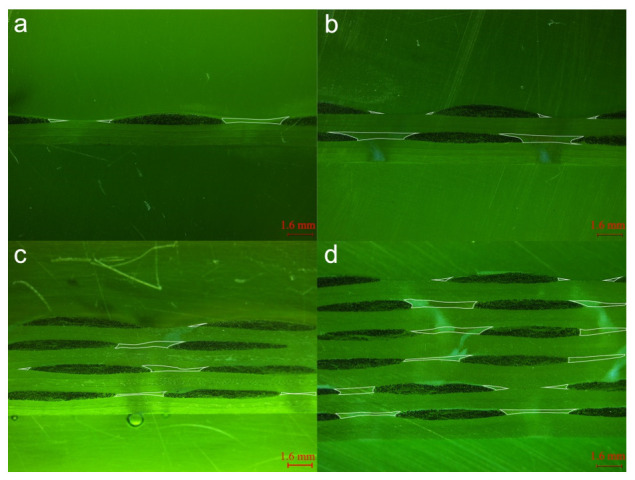
Weft cross-section views of (**a**) 1-layer; (**b**) 2-layer; (**c**) 4-layer; (**d**) 6-layer quadriaxial NCF reinforced laminates.

**Figure 5:**
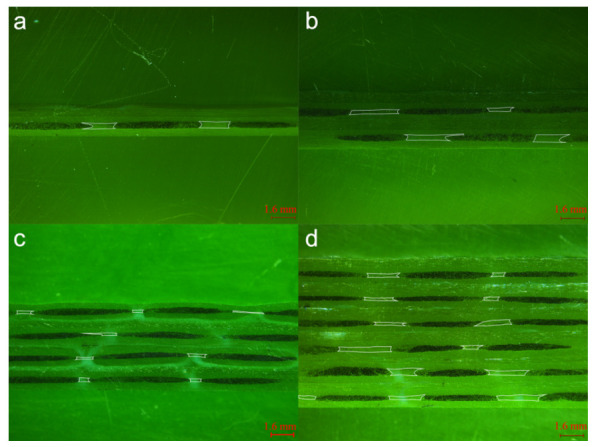
Warp cross-section views of (**a**) 1-layer; (**b**) 2-layer; (**c**) 4-layer and (**d**) 6-layer quadriaxial NCF reinforced laminates.

**Figure 6:**
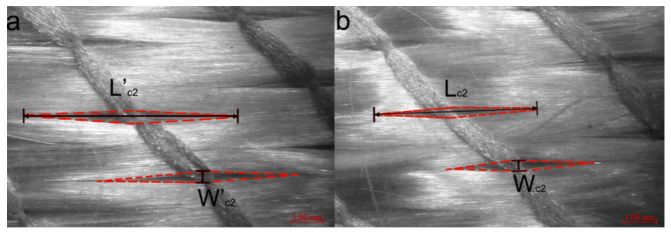
In-plane microstructure of ±45° fiber piles of quasi-axial non-crimp fabric in (**a**) uncompressed state and (**b**) compressed in vacuum pressure.

Images were taken using Leica S ApoE Stereomicroscope and the micrographs were observed by LAS X Live Image Builder software. Meso-channels between 0° tows show distinct characteristics in terms of cross-sectional geometries (Figure 4). The cross-sections of meso-channels in the micrograph are highlighted by white lines. From Figure 4a,b, channels in 1-layer and 2-layer samples exhibit regular and quasi-trapezoidal shapes; whereas the 4-layer and 6-layer samples (Figure 4c,d) contain distorted trapezoidal channels and twisted triangular meso-channels. It is obvious that increasing the number of layers causes distortion of the channels as well as the nesting of ±45° fiber piles, which in turn results in the formation of smaller triangular channels between 0° tows. Meso-channels between 90° tows show similar characteristics in terms of cross-sectional geometries (Figure 5). The cross-sections of meso-channels between 90° tows are highlighted by white lines. Note that the height of channels varies greatly with the increase of compaction level, while minimal fluctuation is observed along the width direction of 90° tows at each multilayer preforms. The paths of 90° tows are mostly straight parallel lines at constant spacing. Figure 6 demonstrates the in-plane microstructure of ±45° fiber piles of quasi-axial non-crimp stitch binded fabric at uncompressed and compressed states under vacuum. For uncompressed fabric, the fibers are fixed by stitch loops and wedge-shaped channels are formed between fibers on the surface of fiber pile. Comparing the dimensions of the wedge-shaped channels at uncompressed and compressed states, the length of the channels (*L’_c_*_2_ to *L_c_*_2_) is reduced at the atmosphere compaction level, while the widest part of the channels (*W’_c_*_2_ = *W_c_*_2_) keeps constant.

### 3.4. Compaction Deformation Mechanism of Quadriaxial NCF Preforms

**Figure 7:**
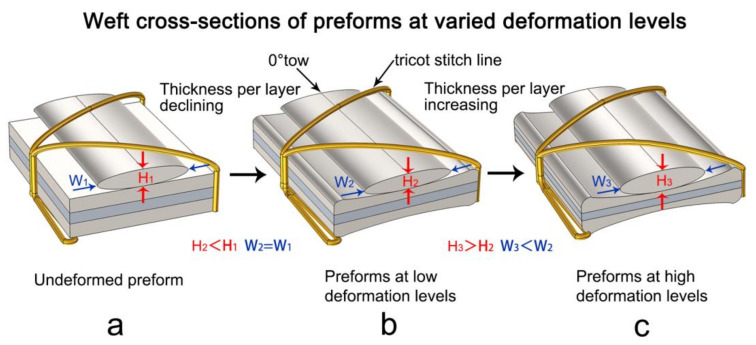
Weft cross-sections of quadriaxial NCF reinforced preforms at varied deformation levels. (**a**) undeformed preform; (**b**) preforms at low deformation levels; (**c**) preforms at high deformation levels.

To verify the conclusions in Section 3.1 and Section 3.2, the internal geometry of quadriaxial preforms was characterized by the vertical cross-section views of laminates. In Figure 4c,d, note that the nesting deformation between layers in the six-layer stack is more pronounced than in four-layer stack. Except that, the height of 0° tows in a six-layer stack is higher and the width of 0° tows is smaller in a six-layer stack. The phenomenon verifies the conclusions in Section 3.1 and Section 3.2, and also reveals the compaction deformation mechanism of multilayered quadriaxial NCF preform in VARTM (Figure 7). It is known that the deformation level increases by increasing the number of reinforced layers. For undeformed preform, the stitch yarn has C-shaped curvature (Figure 7a). Increasing the preform layers, the total fabric thickness is reduced, resulting in the flatten curvature of the stitch yarn at low deformation levels, (Figure 7b). Keeps increasing the preform layers, all the fiber piles except 0° tows are crimped and the part of stitch going across the crimped fiber piles is stretch at high deformation levels. As a result, the part of stitch on the surface of 0° tows shrinks, leaving narrower space along the wideness of 0° tows (Figure 7c). Hence, an increase of the height and a decrease of the wideness of 0° tows are obtained, raising the thickness of quadriaxial NCF fabric. At the same time, the number of flow channels between 0° tows increases by adding more layers and the cross-section of the channels expands by the constraints of stitch yarns, which denotes lower flow resistance, higher permeability and more rapid permeability increasing rate.

## 4. Geometrical Modeling of Quadriaxial NCF Preforms

In a first step, realistic geometrical modeling is applied to fabric layers. The fabric geometry mainly undergoes kinematic changes, i.e., (1) the change of local yarn cross-sections around the yarn axis; (2) nesting deformation of weft backing layers. The deformation is simulated based on a geometrical modeling approach, considering the actual yarn variations in composite laminates.

### 4.1. Modeling the Variations of Local Yarn Cross-Sections

**Figure 8:**
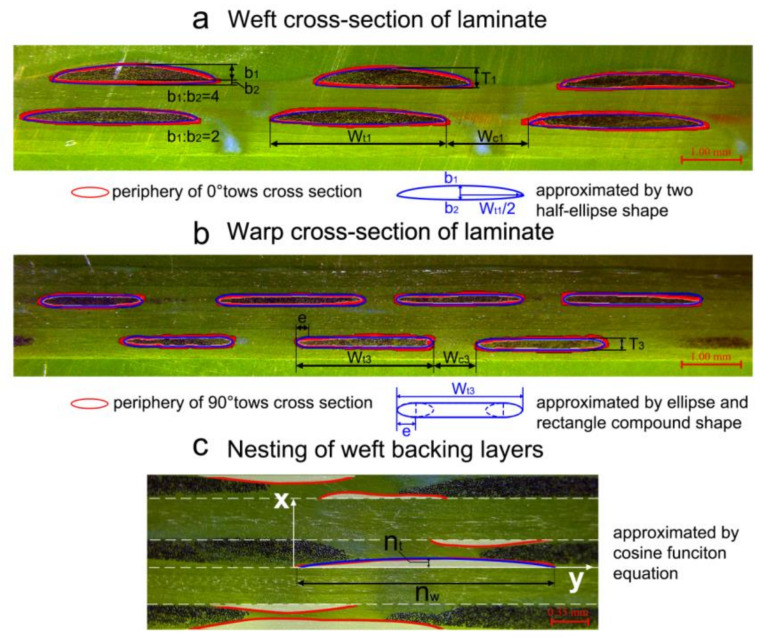
Shapes fitting for (**a**) 0° tows cross-sections; (**b**) 90° tows cross-sections; (**c**) nesting deformation of weft backing layers.

To determine the dimensions of 0° tows cross-section at different compaction levels, the micrographs of 0° tows cross-sections are analyzed (Figure 8a). The cross-sections are approximated by two half ellipse shapes with the same major axis length (*a*_1_) and different minor axis lengths (*b*_1_, *b*_2_). Since the upper fabric surface is covered by a deformable plastic film, the cross-section of 0° tows in the upper fabric is different from others. For the cross-section of tows in the upper layer, the ratio between *b*_1_ and *b*_2_ is four, while for the cross-section of tows in other layers, the ratio between *b*_1_ and *b*_2_ is two. Thirty 0° tows cross-sections were processed for each type of preforms. The average values of the width of the cross-sections (*W_t_*_1_), the width of the gap between 0° tows (*W_c_*_1_*)*, and the height of the cross-sections (*T*_1_) were measured. Similarly, thirty cross-sections of 90° tows for each sample were measured and each individual cross-section is approximated by a compound shape composed of two ellipses and a rectangle (Figure 8b). Average values of the height (*T*_3_) of the fiber pile, the width of the 90° tows (*W_t_*_3_), the width of the gap between 90° tows (*W_c_*_3_) and the major axis length of the ellipse in the compound fitting shape (*e*) for each stack were measured and calculated.

### 4.2. Modeling the Nesting Deformation of Weft Backing Layers

In addition, the nesting effect of fiber piles is characterized. Notice that the ±45° fiber piles have the most obvious nesting deformation and other fiber piles have very small nesting deformation. For simplicity, only the weave paths of ±45° fiber piles are characterized by fitting cosine function equations (Figure 8c). The original position of the ±45° fiber piles is the tangent line at the highest point of the nearest 0° tows cross-section. The maximum vertical displacements *n_t_* and the wideness *n_w_* of the nesting deformation are measured based on the original positions. Considering a nested section at *x* position shifting relative to the original position by *y* (*x*, *y* coordinates are illustrated in Figure 8c). The vertical distance between the surface of ±45° fiber piles in nested section and their original location are described by a cosine function,

(2) $$y=\left|\frac{n_{t}}{2}(\cos\frac{2\pi }{n_{s}}x-1)\right|$$

Specifically, the wedge-shaped channels on +45° pile and −45° pile are identical under vacuum compaction and they are idealized as symmetric diamond shapes as shown in Figure 6b. The average values of width *W_c_*_2_ and the height *L_c_*_2_ of the channels were measured. Along with other parameters mentioned above, the average values and standard deviations are presented in Table 5.

### 4.3. Unit Cell Generation

**Figure 9:**
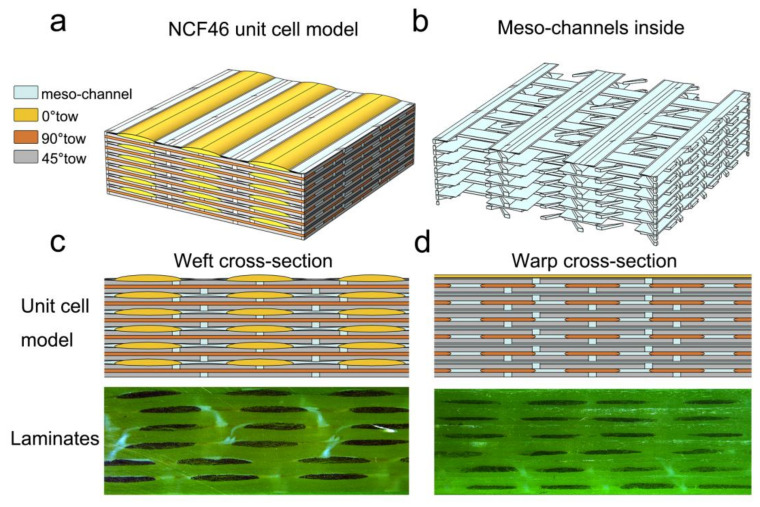
(**a**) Unit cell model of six layers of quadriaxial NCF fabric; (**b**) meso-channel networks inside the unit cell model; (**c**) comparison of cross-sections between the unit cell model and the laminate along warp direction; (**d**) comparison of cross-sections between the unit cell model and the laminate along weft direction.

The modeling approach described in Section 4.1 and Section 4.2 is implemented in the construction of the unit cell models in the modeling tool provided by COMSOL Multiphysics. Unit cell models that consist of one layer, two layers, four layers, and six layers of fabrics are built in the same way. The unit cell domains are defined to ensure the translational periodicity for the fabric layer and the length of all the unit cells *S* is constant. The input parameters for generating all the unit cell models are specified in Table 6. Figure 9 illustrates the unit cell model of six plies of quadriaxial NCF fabrics (NCF46) and the warp/weft cross-sections of the geometrical model. The warp/weft cross-sections of the geometrical model are compared with the warp and weft cross-sections of laminates, demonstrating that the geometrical model can accurately describe the actual fibrous structure of the multilayered preform.

## 5. Permeability Computation

### 5.1. Fluid Flow Models

A volume-averaging method is used to evaluate the permeability in the unit cells. The resin filtration process is considered as the flow of a Newtonian fluid in a dual-scale porous medium, consisting of the channel network between the inter-tow and intra-tow regions [22,36,37,38]. The equivalent in-plane permeability can be determined by substituting the resulted volume–average pressure fields and volume average velocity fields into Darcy’s law (Equations (3) and (4)).

(3) $$u_{out}=\frac{1}{\rho A}\left(\int_{Vmeso}^{0}M_{meso}dv+\int_{Vmicro}^{0}M_{micro}dv\right)$$

(4) $$K_{eq}=\frac{u_{out}\mu L}{\Delta P}$$

where *u_out_* is the volume average velocity, *M_meso_* and *M_micro_* represent the mass of the flow in the inter-tow region and intra-tow region, *ρ* is the density of the resin and *A* is the cross-section area of the flow outlet. Brinkman equation (Equation (5)) and continuity equation (Equation (6)) are applied to model the flow impregnation process in both the inter-tow and intra-tow region [21,39,40].

(5) $$\mu \nabla^{2}u-\nabla P-\mu K_{Br}^{-1}\cdot u=0$$

(6) ∇⋅u=0

where *K_Br_* denotes the equivalent permeability of the intra-tow or inter-tow region, *u*, *µ*, *∇P* are the volume-averaged velocity, the dynamic fluid viscosity, the pressure gradient across the porous medium, respectively. In the Brinkman equation, the equivalent permeabilities of intra-tow and inter-tow flow domains are required. Previous analytical methods are used to characterize the equivalent permeability values of intra-tow or inter-tow domains. For the intra-tow domains, it is assumed that the fiber bundle consists of parallel impermeable filaments arranged in a hexagonal array with periodic patterns. The permeability for flow parallel or transverse to the fibers is determined by Gebart equations (Equations (7) and (8)) [41,42,43,44].

(7) $$K_{micro}^{∥}=\frac{8R^{2}}{C_{1}}\frac{{\left(1-V_{f}\right)}^{3}}{V_{f}^{2}}$$

(8) $$K_{micro}^{⊥}=C_{2}{\left(\sqrt{\frac{V_{f\max}}{V_{f}}-1}\right)}^{2.5}R^{2}$$

where *K*^‖^*_micro_* and *K*^⊥^*_micro_* are the longitudinal and transverse permeabilities of the tows, *C*_1_ = 53, *C*_2_ = $16/(9\pi \sqrt{6})$ and *V_fmax_* = $\pi /2\sqrt{3}$ for hexagonal fiber arrangement. The average fiber radius *R* and fiber volume fraction *V_f_* can be derived from micrographs of laminate cross-sections. For tows that orientating *θ* degree relative to the flow direction, the effective permeability on the flow direction can be predicted based on the principal permeability *K*^‖^*_micro_* and *K*^⊥^*_micro_*. As the angle *θ* between principle permeability and flow direction is equal to 45°, the permeability along the flow direction *K^θ^_micro_* is estimated as [45],

(9) $$K_{micro}^{\theta }=\frac{K_{micro}^{∥}+K_{micro}^{⊥}}{2}$$

**Figure 10:**
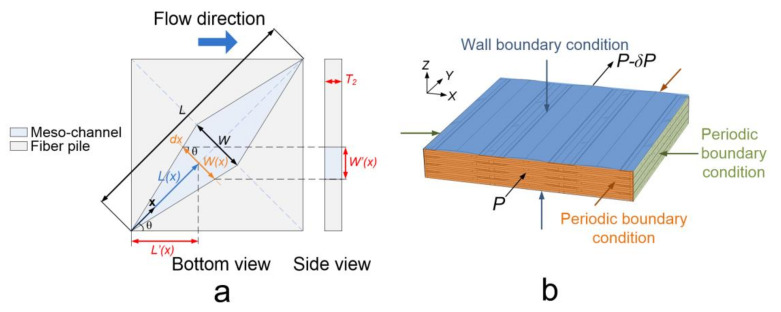
(**a**) Solution of the effective permeability of a wedge-shaped channel which oriented *θ* from the flow direction (*θ* = 45°) (**b**) boundary conditions assigned in the unit cell model.

Here the effective permeability of inter-tow gaps between 0° tows and 90° tows with irregular cross-section can be predicted by the perimeter and the area of the cross-section [13,36],

(10) $$K_{meso}=\frac{A_{c}^{2}}{2P_{c}^{2}}$$

where *P_c_* and *A_c_* are the perimeter and area of the meso-channel cross-section, respectively and the effective permeability of each channel is calculated in Matlab using the perimeter and area measurements obtained in Section 4. As for the wedge-shaped meso-channels in ±45° fiber pile, the permeability of an individual meso-channel is calculated based on the analytical solution of duct flow. Note that the effective permeability in a duct channel could be expressed as [46],

(11) $$K_{e}=\frac{W^{2}}{12}\left[1-\frac{192W}{\pi^{2}T}\sum_{i=1,3,5\ldots }^{\infty }\frac{\tanh(\frac{i\pi T}{2W})}{i^{5}}\right]$$

where *K_e_* is the equivalent permeability, *T* is the depth of the meso-channel, and *W* is the width. Hence, it is inferred that the equivalent permeability of a meso-channel with rectangular cross-sections could be calculated from the width and the depth of the cross-section. In this case, a wedge-shaped channel that oriented *θ* from the flow direction (*θ* = 45°) is presented with a constant height *T*_2_, length *L* and maximum width *W* (Figure 10a). The meso-channel is considered as an infinite amount of serially connected channels of length *dx* with a spatial dependent cross-sectional area *Ax*. Supposing a minor meso-channel of length *dx* locates at *x*, the effective permeability of the minor meso-channel along the flow direction can be estimated from the width of the cross-section *W’*(*x*) and the depth of the channel *T*_2_ by Equation (11). Also, the distance from the resin inlet boundary to the minor channel along the flow direction is defined as *x’*. Notice that the effective permeability of the minor channel is a function of *x*. Relations between *W*(*x*), *W’*(*x*), *x* and *x’* can be expressed in Equations (12)–(14). Afterward, from the integration of all the connected minor channels along the flow direction, the effective permeability of the entire meso-channel on the flow direction can be determined by Equation (15).

(12) x′=xcosθ

(13) W(x)′=W(x)cosθ

(14) $$W\prime (x)=\frac{2W}{L}x\cos\theta$$

(15) $$K_{meso}^{\theta }=L\cos\theta {\left(\int_{0}^{L\cos\theta }\frac{1}{K(x)}dx\right)}^{-1}$$

where *K*(*x*) is the effective permeability of the minor channel with length *dx*, *K^θ^_meso_* is the effective permeability of the entire meso-channel on the flow direction. Therefore, all the inter-tow and intra-tow permeabilities are estimated and the values assigned in the flow models are presented in Table 7.

### 5.2. Boundary Conditions

The boundary conditions assigned in the unit cell model is illustrated in Figure 10b. A pressure gradient (500 Pa) is applied to the inlet and outlet boundaries of the unit cell. At the boundaries between the inter-tow domain and intra-tow domain, slip boundary conditions are imposed. In the X and Y direction (in-plane directions), periodic boundary conditions are used and along the Z direction (through the thickness direction), wall boundary conditions are justified.

## 6. Results and Discussion

**Figure 11:**
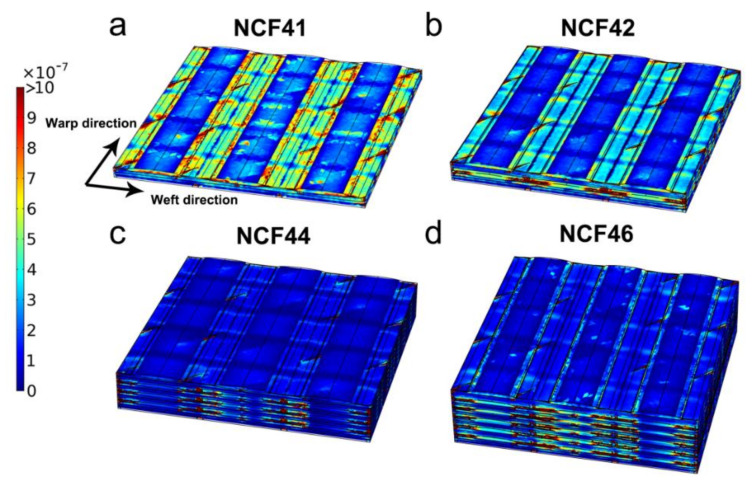
Flow velocity magnitudes in unit cells predicted by simulations.

The simulation is carried out in Free and Porous Media Flow Module of COMSOL Multiphysics using the stationary nonlinear solver. The mesh consists of 626,370 to 2,188,859 tetrahedral elements for different unit cell geometries. The predicted velocity profiles of quadriaxial NCF with multilayered are derived from the numeric simulations (Figure 11). In general, the numerical modeling enables a direct relationship between the flow velocity and the flow in meso-channels between 0° tows along warp direction. And, the flow velocity at other regions (intra-tow region and other smaller inter-tow channels) is much lower than the velocity of the flow in meso-channels between 0° tows. However, due to the different nesting and compaction behavior in the unit cells, the flow resistance of the intra-tow region and other smaller inter-tow channels greatly varied. Compared to its results with the experimental data reported in Section 2.3, the reliability of the proposed finite element model is validated. For the preforms in this study, standard deviations of experimental data and numerical predictions are listed in Table 8. The uncertainties of the predicted permeability obtained by the numerical method by comparison with experimental results are estimated to be 12.8%, 13.8%, 14.4% and 9.4%, respectively. Also, the permeability scatter in predicted values increases with the layers increasing and the same trend is observed in the scatter distribution.

**Figure 12:**
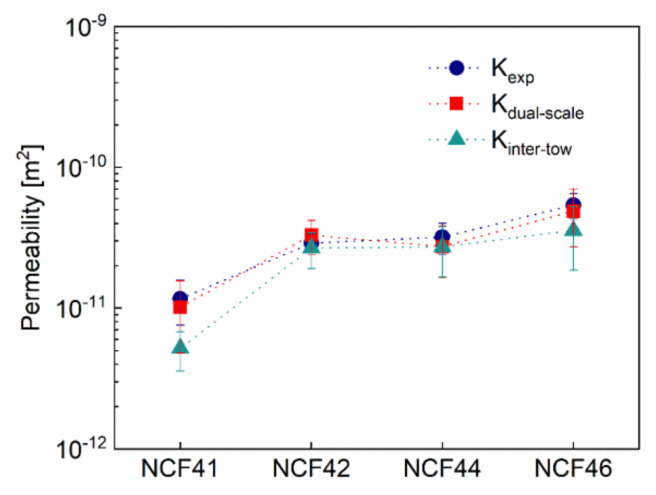
Comparison of the permeability values derived from experimental tests, prediction values considering dual-scale flow and prediction values considering only inter-tow flow.

Figure 12 compares the permeability values derived from experimental tests *K_exp_*, numerical computation results considering dual-scale flow *K_dual-scale_* and the results considering merely inter-tow flow *K_inter-tow_*. The error bars represent the permeability variations due to the geometrical measurements. Note that the *K_dual-scale_* agrees well with *K_exp_* and the variation of *K_dual-scale_* is similar to the range of experimental *K_exp_*. The deviation between *K_dual-scale_* and *K_exp_* can be attributed to the random variability in the spatial distribution of the tows and the irregular arrangement of the fibers, which is not described in the geometrical models. Moreover, it reveals that as the fiber volume fraction increases, the intra-tow flow have less effect on the in-plane permeability becomes insignificant. When the fiber volume fraction of preform exceeds 55%, the influence of intra-tow flow on the in-plane permeability can be neglected.

## 7. Effects of Structural Parameters on the In-Plane Permeability

**Figure 13:**
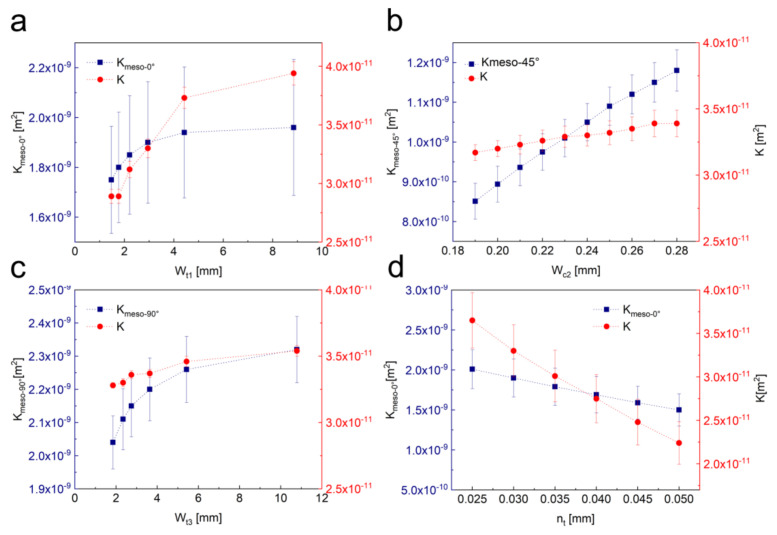
Effect of fabric structural parameters on in-plane permeability. (**a**) width of 0° tows; (**b**) width of 90° tows; (**c**) width of the wedge-shaped channel in ±45° fiber piles; (**d**) maximum vertical displacement of ±45° fiber piles in the nested section.

It is crucial to investigate the effect of structural parameters on the in-plane permeability of fabrics while the fiber volume fraction of the preform remains constant. Here, the influence of the width of 0° tows (*W_t_*_1_), 90° tows (*W_t_*_3_), the width of the wedge-shaped channel in ±45° fiber piles (*W_c_*_2_) and the vertical displacement (*n_t_*) of the nested section on the flow resistance are investigated. In the paramedic study, to keep the fiber with different orientations at a constant fiber volume fraction in the geometrical model, the tows arrangements are varied accordingly. The computed values of meso-channel permeability, global permeability and the deviations due to geometrical measurements for preform NCF42 are plotted as a function of *W_t_*_1_, *W_t_*_3_, *W_c_*_2_ and *n_t_* (Figure 13). It can be seen that a slight change in the fabric structure will cause a significant change in the equivalent permeability of the meso-channel. In Figure 13a, when the fiber volume fraction of 0° tows remains constant, the wider of 0° tows, the higher the global permeability, but the increasing rate will gradually decrease. The same trend is observed by increasing the width of 90° tows. However, when the width of 0° tows is increased by two millimeters, the increasing rate of global permeability can be up to 32%; while the width of 90° tows is increased by two millimeters, the increasing rate of global permeability is merely 2.9%. For the meso-channels in ±45° fiber piles, expanding the width of the channel by one millimeter, the increment in global permeability is 6.9%. Additionally, if the vertical displacement of ±45° fiber piles is increased by 0.02 mm in the nested section, the global permeability is reduced by 32%. Therefore, for a typical quasi-axial NCF, if the fiber volume fraction and the stitch distance remain constant, in order to increase the permeability of the fabric, the nesting deformation of weft backing layers should be avoided and the tex of the tows along flow direction should be designed as large as possible. In addition, the tension of the stitch yarn can be increased in the allowable range to widen the meso-channels in weft backing layers to enhance the fabric global permeability. However, actually, at a constant fiber volume fraction, wider tows in the fabric bring wider gaps between them. And, this always induces a higher level of nesting deformation to the neighbor layers. Together with the bending stiffness of the neighbor fiber piles, this numerical prediction methodology can be applied to precisely predict the permeability and used in the design of quadriaxial NCF fabrics.

## 8. Conclusions

Flow and compaction properties were investigated for preforms reinforced by multilayered biaxial NCF and quadriaxial NCF using VARTM. Compression responses and internal geometry characterization on the ply level showed that the degree of nesting between quadriaxial NCF layers is more obvious and the nesting deformation between layers of quadriaxial NCF was affected by the interaction with stitch yarns. Due to the meso-channels in the fibrous structure and the nesting between layers, the in-plane permeability of quadriaxial NCF did not follow an inverse proportional relationship with the fiber volume fraction. Meanwhile, a rapid increase of permeability and a drop of fiber volume fraction happened as the increases of layers. According to the compaction experiments and geometrical measurements of preform structures, tows cross-section variations and nesting deformations were modeled to simulate the realistic structure of the preforms. The comparison of permeability values predicted based on flow simulations using these detailed geometrical models with corresponding experimental data indicated a maximum difference of 14.4% for preforms with varied lay-ups. Considering that, the predictions shown good accuracy, and, the simulation results also revealed that the intra-tow flow has an obvious influence on the in-plane permeability at low fiber volume fraction. In addition, the correlation between the in-plane permeability and the structural parameters of the quadriaxial NCF at constant fiber volume fraction was investigated. This paper unveiled the compaction mechanism of quadriaxial NCF and verified the feasibility of the numerical modeling approach for permeability predictions of quadriaxial NCF. Moreover, it provided insights to develop quasi-axial NCF reinforcements with high permeability, which is beneficial for the design of multi-axial NCF reinforcements in the future.

## Figures and Tables

**Table 1:** Geometric parameters of quadriaxial NCF (E-DBLT800-6).

Fiber System	Fiber Orientation	Geometry	Areal Weight (g·m^−2^)	Density (Tows·10 cm^−1^)	Linear Density (tex)	Glass Density (g/cm^3^)
1st fiber pile	0°	unidirectional tows	212	25	900	2.54
2nd fiber pile	−45°	weft backing fiber pile	200	–	–	2.54
3rd fiber pile	90°	unidirectional tows	201	35	500	2.54
4th fiber pile	45°	weft backing fiber pile	200	–	–	2.54

**Table 2:** Geometric parameters of biaxial NCF (E-DB800).

Fiber System	Fiber Orientation	Geometry	Areal Weight (g·m^−2^)	Density (Tows·10 cm^−1^)	Linear Density (tex)	Glass Density (g/cm^3^)
1nd fiber pile	−45°	unidirectional tows	401	40	700	2.54
2th fiber pile	45°	unidirectional tows	401	40	700	2.54

**Table 3:** Stitching parameters of quadriaxial NCF and biaxial NCF.

Fabric	Stitch Pattern	Stitch Distance D_x_ (mm)	Stitch Length D_y_ (mm)
E-DBLT800-6	Tricot loop	4.16 ± 0.12	2.5 ± 0.11
E-DB800	Chain loop	5.25 ± 0.14	2.5 ± 0.09

**Table 4:** Layer numbers and stacking sequences of the preforms used in experiments.

Test	Fabric	Layups	Stacking Sequences
NCF21	E-DB800	1	0
NCF22	E-DB800	2	[0] ^2^
NCF24	E-DB800	4	[0] ^4^
NCF26	E-DB800	6	[0] ^6^
NCF41	E-DBLT800-6	1	0
NCF42	E-DBLT800-6	2	[0] ^2^
NCF44	E-DBLT800-6	4	[0] ^4^
NCF46	E-DBLT800-6	6	[0] ^6^

**Table 5:** Geometry parameters measured for the quadriaxial NCF preforms composed of multi layers.

Geometry Parameters of Different Preforms	NCF41	NCF42	NCF44	NCF46
Width of 0° tows *W_t_*_1_ (mm)	2.99 ± 0.06	2.86 ± 0.16	2.95 ± 0.14	2.71 ± 0.15
Width of the meso-channel between 0° tows *W_c_*_1_ (mm)	1.49 ± 0.21	1.36 ± 0.19	1.26 ± 0.10	1.54 ± 0.28
Height of 0° tows *T*_1_ (mm)	0.289 ± 0.008	0.267 ± 0.03	0.202 ± 0.03	0.275 ± 0.02
Maximum Vertical displacement of ±45° fiber piles due to nesting *n_t_* (mm)	0.06 ± 0.01	0.025 ± 0.01	0.03 ± 0.02	0.04 ± 0.02
Horizontal spread width of the nested section *n_w_* (mm)	1.42 ± 0.21	1.26 ± 0.19	1.16 ± 0.10	1.45 ± 0.28
The width of the meso-channels on ±45° fiber piles *W_c_*_2_ (mm)	0.24 ± 0.01	0.25 ± 0.01	0.23 ± 0.02	0.24 ± 0.01
The height of the meso-channels on ±45° fiber piles *L_c_*_2_ (mm)	2.74 ± 0.20	2.8 ± 0.15	2.62 ± 0.20	2.76 ± 0.31
Height of ±45° fiber piles *T*_2_ (mm)	0.189 ± 0.025	0.185 ± 0.02	0.162 ± 0.04	0.162 ± 0.05
Width of 90° tows *W_t_*_3_ (mm)	2.34 ± 0.12	2.05 ± 0.185	2.39 ± 0.23	2.15 ± 0.18
Width of the meso-channel between 0° tows *W_c_*_3_ (mm)	1.03 ± 0.14	1.37 ± 0.08	1.15 ± 0.17	1.28 ± 0.23
Major axis length of the ellipse in the compound fitting shape *e* (mm)	0.15	0.15	0.2	0.2
Height of 90° tows *T*_3_ (mm)	0.166 ± 0.013	0.138 ± 0.023	0.126 ± 0.018	0.126 ± 0.025
The fiber volume fraction of tows *V_f-tow_*	0.5 ± 0.02	0.54 ± 0.02	0.63 ± 0.03	0.58 ± 0.03
Fiber diameter (μm)	17 ± 3	17 ± 3	17 ± 3	17 ± 3

**Table 6:** Input parameters for generating unit cell models for each preforms.

Unit Cells of Different Preforms	NCF41	NCF42	NCF44	NCF46
S (mm)	13.5	13.5	13.5	13.5
W_t1_ (mm)	2.95 ± 0.15	2.95 ± 0.15	2.95 ± 0.15	2.71 ± 0.15
W_c1_ (mm)	1.55 ± 0.15	1.55 ± 0.15	1.55 ± 0.15	1.79 ± 0.15
T_1_ (mm)	0.289	0.267	0.202	0.275
n_t_ (mm)	0.06 ± 0.01	0.03 ± 0.01	0.03 ± 0.02	0.05 ± 0.02
n_w_ (mm)	1.55 ± 0.15	1.55 ± 0.15	1.55 ± 0.15	1.79 ± 0.15
W_c2_ (mm)	0.24	0.24	0.24	0.24
L_c2_ (mm)	2.74	2.74	2.74	2.74
T_2_ (mm)	0.189	0.185	0.162	0.162
W_t3_ (mm)	2.34	2.34	2.34	2.34
W_c3_ (mm)	1.035	1.035	1.035	1.035
e (mm)	0.15	0.15	0.2	0.2
T_3_ (mm)	0.166	0.138	0.126	0.126
V_f-tow_	0.5	0.54	0.63	0.58
Fiber diameter(μm)	17	17	17	17

**Table 7:** Intra-tow permeability and inter-tow permeability assigned in unit cell models.

Unit Cells of Different Preforms	K_micro-0°_ (m^2^)	K_meso-0°_ (m^2^)	K_micro-45°_ (m^2^)	K_meso-45°_ (m^2^)	K_micro-90°_ (m^2^)	K_meso-90°_ (m^2^)
NCF41	5.45*10^−12^	1.63*10^−^^9^ ± 3.30*10^−^^10^	3.32*10^−^^10^	1.08*10^−^^9^	1.18*10^−^^12^	3.01*10^−^^9^
NCF42	3.64*10^−12^	2.11*10^−^^9^ ± 4.50*10^−^^10^	2.22*10^−^^12^	1.05*10^−^^9^	7.95*10^−^^13^	2.11*10^−^^9^
NCF44	1.39*10^−12^	9.35*10^−^^9^ ± 3.49*10^−^^10^	8.45*10^−^^13^	8.98*10^−^^10^	2.98*10^−^^13^	1.73*10^−^^9^
NCF46	2.40*10^−12^	2.28*10^−^^9^ ± 6.60*10^−^^10^	1.46*10^−^^12^	8.98*10^−^^10^	5.24*10^−^^13^	1.73*10^−^^9^

**Table 8:** Comparison of permeability calculated from simulations and permeability tested in experiments.

Preforms	Permeability Tested in Experiment /m^2^	Predicted Permeability/m^2^	Error/ |Kpredicted-Kexperiment|/Kexperiment
NCF41	1.17*10^−1^^1^ ± 4.09*10^−1^^2^	1.02*10^−1^^1^±5.40*10^−1^^2^	12.8%
NCF42	2.9*10^−1^^1^ ± 5.09*10^−1^^2^	3.30*10^−1^^1^± 9.10*10^−1^^2^	13.8%
NCF44	3.2*10^−1^^1^ ± 8.13*10^−1^^2^	2.74*10^−1^^1^ ± 1.09*10^−1^^1^	14.4%
NCF46	5.4*10^−1^^1^ ± 1.11*10^−1^^1^	4.89*10^−1^^1^ ± 2.16*10^−1^^1^	9.4%
